# A Sustainable Combined Approach to Control the Microbial Bioburden in the School Environment

**DOI:** 10.3390/microorganisms13040791

**Published:** 2025-03-30

**Authors:** Maria D’Accolti, Irene Soffritti, Eleonora Mazziga, Francesca Bini, Matteo Bisi, Antonella Volta, Sante Mazzacane, Elisabetta Caselli

**Affiliations:** 1 Section of Microbiology, Department of Environmental Science and Prevention, University of Ferrara, 44121 Ferrara, Italy; irene.soffritti@unife.it (I.S.); eleonora.mazziga@unife.it (E.M.); francesca.bini@unife.it (F.B.); 2 CIAS Research Centre, University of Ferrara, 44122 Ferrara, Italy; matteo.bisi@unife.it (M.B.); antonella.volta@unife.it (A.V.); sante.mazzacane@unife.it (S.M.)

**Keywords:** school environment, pathogen bioburden, probiotic-based sanitation, green interventions

## Abstract

The indoor microbiome is a dynamic ecosystem including pathogens that can impact human health. In this regard, the school environment represents the main living space of humans for many years, and an unhealthy environment can significantly condition students’ health. School rooms can suffer from insufficient ventilation and the use of building materials that may favor pathogen contamination, mostly sanitized by conventional chemical-based methods, which can impact pollution, have temporary effects, and induce the selection of antimicrobial resistance (AMR) in persistent microbes. In the search for sustainable and effective methods to improve the healthiness of the classroom environment, a pre–post case–control study was performed in an Italian high school. Over a year, different interventions were sequentially placed and evaluated for their impact on bioburden and air quality, including the introduction of plants, a mechanical ventilation system, and probiotic-based sanitation (PBS) in substitution for chemical sanitation. Through continuous microbial monitoring of the enrolled school rooms, via culture-dependent and -independent methods, a remarkable bioburden level was detected at baseline (around 12,000 and 20,000 CFU/m^2^, before and after classes, respectively), composed mostly of *Staphylococcus* spp. and fungi. Some decrease in fungal contamination was observed following the introduction of plants. Still, the most significant decrease in pathogens and associated AMR was detected following the introduction of ventilation and PBS, which decreased pathogen level by >80% (*p* < 0.001) and AMR by up to 3 Log_10_ (*p* < 0.001) compared to controls. Collected data support the use of combined strategies to improve indoor microbial quality and confirm that PBS can effectively control bioburden and AMR spread not only in sanitary environments.

## 1. Introduction

The environmental microbiome is a key factor for human health within built environments (BEs) [1,2,3]. BEs are complex organic systems because of the numerous interdependent microbial communities they host, which have a significant impact on human health and potentially threaten their well-being [4]. Educational institutions, such as schools, serve as primary living environments during the educational phases of human life, as students typically spend one-third of their day there. Characterizing the microbiome in these environments is crucial to understanding how it can affect students’ health [5], well-being, and daily performance. Indoor school microbiomes can be shaped by several factors, including occupants’ features (gender, age groups) and activity [6,7], ventilation modalities [8], building materials [9], air pollutants [5], and environmental hygiene practices [10]. There is evidence that indoor air quality (IAQ) and ventilation in school buildings play a crucial role in spreading bacterial and fungal aerosol contamination, leading to an increased risk of developing sick building syndrome (SBS), characterized by mucous membranes and skin irritation, mental fatigue, nausea, and respiratory issues, including asthma and rhinitis [2,11,12,13]. In European schools, naturally ventilated old buildings often display inadequate ventilation, and insufficient airflow can lead to poor air quality, elevated CO_2_ levels, and worsened health issues associated with exposure to indoor pathogen microbes [14,15]. Controlled mechanical ventilation generally can improve indoor air quality, as judged by monitoring CO_2_ and other pollutants [8,16], but its impact on the indoor environmental microbiome remains unclear. Besides, indoor building materials and architectural designs can also have an impact on the BE microbiome, including the school one [9]. Materials that retain moisture, such as paper and fabrics, can indeed promote microbial growth, particularly of pathogenic fungi that can potentially trigger allergic reactions or respiratory diseases, including the already mentioned SBS [17,18].

To promote a healthier school environment, the “naturalizing” concept—introducing plants and creating “green” spaces—has recently gained interest, as natural elements have been associated with many benefits for students, including improvements in physical and mental health [19]. Indoor plants can act as a source of microbial dispersal in indoor environments [20], including beneficial microorganisms that can positively impact students’ health [21]. For instance, *Epipremnum aureum* (*Golden pothos*), a widely popular houseplant commonly used for ornamental purposes, has been shown to offer health-related benefits in school environments, such as improving air humidity, temperature, and CO_2_ levels, despite no significant impact on airborne molds being evidenced [22].

Surface sanitation is another crucial factor in keeping the school environment healthy and avoiding pathogen spread [23]. High-touch surfaces (such as desks, floor, and door handles) can be reservoirs of potentially dangerous microbes, including *Staphylococcus* spp., that can cause a range of infections from mild skin conditions to more severe, life-threatening illnesses [24,25,26]. Daily cleaning and disinfection of classrooms and common areas can help reduce the spread of pathogens and associated diseases [23]. However, conventional sanitation methods often involve harsh chemical disinfectants, which, although effective in the short term, have limited long-lasting effects and contribute to pollution [27,28]. Moreover, chemical disinfectants also eliminate beneficial microbes, which are important to maintaining a high level of biodiversity and the eubiosis of indoor environments [29]. Recently, innovative sanitation systems based on the use of eco-friendly detergents containing safe *Bacillus* probiotics have been reported to be highly effective in controlling the bioburden in sanitary environments, while also preventing the increase in AMR and pollution [30,31,32,33]. A system named PCHS (Probiotic Cleaning Hygiene System), including the species *Bacillus subtilis*, *Bacillus velezensis* (ex *pumilus*), and *Priesta megaterium* (ex *B. megaterium*), was shown to stably reduce pathogens and hospital infections [34,35] via stable modulation of the indoor microbiome and associated AMR [34,35,36,37,38,39,40,41,42,43].

Based on the reported observations and experimental data, the present study aimed to investigate the impact of integrated sustainable applications, including the introduction of plants, ventilation, and PCHS sanitation, on the indoor microbiome in an Italian school, with the goal of creating a healthier environment for students and teachers.

## 2. Materials and Methods

### 2.1. Study Design

A pre–post case–control study was conducted at the High School “Liceo Ludovico Ariosto” in Ferrara, Italy, from July 2023 to May 2024. The study represented a part of a Path for Transversal Skills and Orientation (PTSO) project, approved by the school director. Two classrooms with a 48.7 m^2^ surface and superimposable features were enrolled in the study, one of which was subjected to experimental interventions (test room, TR) while the other did not receive any interventions and served as a control (control room, CR). The main characteristics of the classrooms are summarized in Table 1. Both of them were equipped with large windows that faced outward, providing natural light and ventilation. Extra windows were located in the ceiling at 5.15 m of height, and additional windows facing the main corridor were positioned opposite the outward windows, ensuring horizontal airflow in the room. The classroom was equipped with 17 student tables and one teacher’s desk.

The study included four subsequent phases: phase T0 (5 months, July 2023 to November 2023), during which usual conventional chemical-based sanitation via alcohol-based products and natural ventilation via window opening were applied; phase T1 (1 month, December 2023), during which specific plants were placed in the TR, maintaining conventional sanitation and ventilation systems; phase T2 (1 month, January 2024), during which a mechanical air ventilation system was added to the TR, together with plants and maintaining conventional sanitation; and phase T3 (4 months, February 2024 to May 2024), during which the TR received probiotic-based sanitation in substitution of the conventional chemical-based one, in addition to plants and mechanical ventilation. The CR did not receive any interventions and was surveyed as a control throughout the whole study period.

### 2.2. Green Interventions: Plants, Ventilation, and Probiotic-Based Sanitation

Three subsequent interventions were implemented in the TR to improve the indoor environment quality: placement of green plants (T1), installation of a mechanical ventilation system (T2), and implementation of probiotic-based sanitation in substitution of the conventional chemical-based one (T3).

The plant species placed in the TR during the T1 period were *Golden pothos* and *Tillandsia kammii Rauh*. They were chosen for their resilience, their suitability for greening walls, and their ease of manipulation. Students could take care of them and eventually propagate them as part of the PTSO project. The Botanical Garden of the University of Ferrara provided all the plants that stayed in place in the TR until the end of the study.

The mechanical ventilation system installed in the TR during the T2 period was the vertically designed VEX 380S (Aldes, Modena, Italy), which had a maximum airflow of 1000 m^3^/h and also included a fan coil unit for heating.

**Figure 1:**
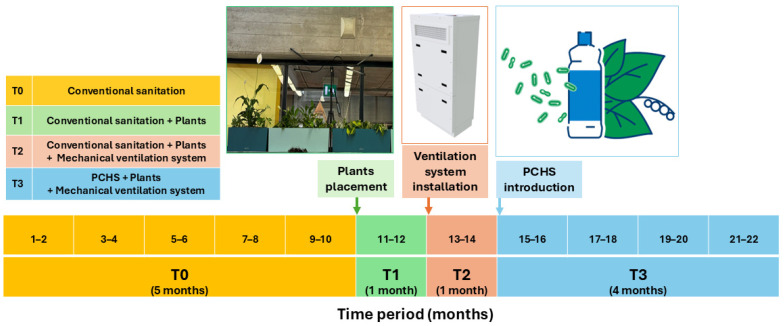
Study design. The study included two classrooms (test room, TR; and control room, CR) and consisted of four subsequent phases: T0 (yellow), a 5-month period with conventional sanitation and natural ventilation; T1 (green), a 1-month period during which plants were placed in the TR; T2 (pink), a 1-month period during which mechanical ventilation was introduced in the TR; and T3 (light blue), a 4-month period during which PCHS was implemented in the TR. The CR did not receive any interventions and served as a control. Samplings were performed bimonthly in the TR and CR, and the number is indicated in the period box.

The probiotic-based cleaning procedure, implemented in the TR during the T3 period, consisted of the Probiotic Cleaning Hygiene System (PCHS, Copma, Italy) [34,36,39], based on an eco-friendly detergent including 10^7^/mL spores of three species of *Bacillus* probiotics (*B. subtilis*, *B. velezensis* ex *B. pumilus*, and *Priesta megaterium* ex *B. megaterium*). It replaced the daily sanitation performed by alcohol-based products. Both conventional and PCHS sanitations were carried out daily at the end of morning lessons by adequately trained school operators, according to standard procedures [39]. The study design and timing are schematically summarized in Figure 1.

### 2.3. Environmental Monitoring

The classroom environment was monitored in continuum throughout the whole study. The environmental parameters assessed included temperature (°C), relative humidity (RH), and CO_2_. The measurements were performed using Testo 160 IAQ Wi-Fi Data Loggers (Testo S.p.A, Milan Italy), providing continuous measurement of the indicated parameters through two sensors positioned in the enrolled classrooms.

**Figure 2:**
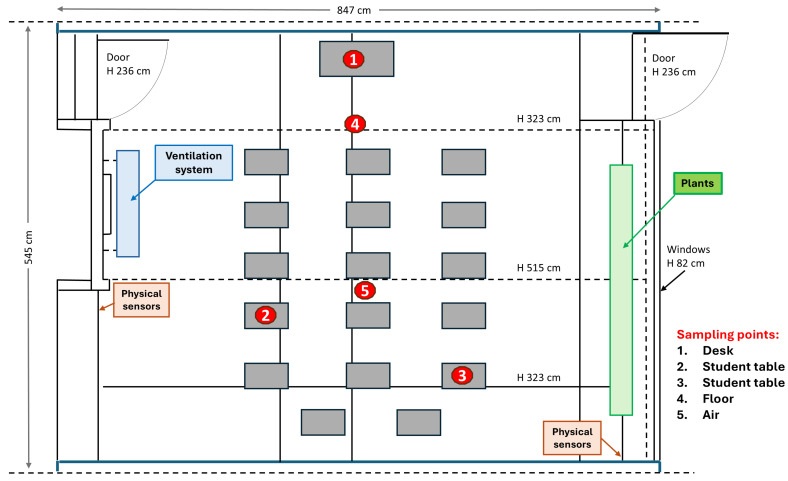
Floor plan of the enrolled classrooms, including sampling point locations.

Microbial monitoring was performed bimonthly during each phase, for a total of 22 sampling campaigns in the whole study period, 10 of which were during T0, 2 of which were during T1, 2 of which were during T2, and 8 of which were during T3. At each sampling campaign, surface and air samples were collected both before and after students’ lessons to evaluate the impact of the students’ presence on microbial contamination. Surface samples included the floor, the teacher’s desk, and two student tables (Figure 2). All surface points were simultaneously sampled by two different methods according to the subsequent type of analysis.

For microbiological analyses, samples were collected in duplicate by using RODAC contact plates 55 mm diameter (corresponding to a 24 cm^2^ surface) containing the following general and specific culture media: plate count agar (PCA, Biolife, Monza, Italy) for the total bacterial count, Baird Parker agar (BP, Sharlab, Milan, Italy) for *Staphylococcus* and *Bacillus* spp., Mac Conkey agar (MCA, Sharlab, Milan, Italy) for *Enterobacteriaceae*, cetrimide agar (CA, Sharlab, Milan, Italy) for *Pseudomonas* spp., and dichloran glycerol agar (DG18, Sharlab, Milan, Italy) for the total fungi, including *Candida* and *Aspergillus* spp. Air sampling was performed at the center of the room via the Surface Air System (SAS) instrument (VWR International, Milan, Italy), collecting 1 m^3^ of air on the same media used for surface sampling. For molecular analyses, duplicate samples were collected from the same surface points by sterile rayon swabs rubbed on a 100 cm^2^ area and then placed in 5 mL of sterile tryptone soy broth (TSB, Biolife, Monza, Italy), as previously described [34,36,39]. All collected samples were immediately refrigerated at 2–10 °C and transported to the laboratory within 12 h. The schematic plan of the classroom and the sampling points are shown in Figure 2.

### 2.4. Microbiological Analyses

Microbiological analyses were carried out on the collected RODAC plates by appropriate incubation and colony-forming unit (CFU) count. Briefly, bacteria were incubated at 37 °C for 24 h or 48 h on general or selective media, respectively. Fungi were incubated at 25 °C for 72 h. At the end of incubation, microbial growth was determined by CFU enumeration. Overall, a total of 2688 microbiological samples were collected from surfaces and the air, including 1280 samples collected during T0, 256 during both T1 and T2, and 896 during T3.

### 2.5. Molecular Analyses

Molecular analyses were performed on the swabs collected in TSB broth. Samples were incubated at 37 °C for 24 h, and microbes were collected by centrifugation (12,000× *g* for 5 min at 4 °C). Total DNA was extracted from the pelletized microbes by a commercial kit (Exgene Cell SV mini kit, Gene All, Seoul, South Korea), following the manufacturer’s instructions. Extracted DNA was quantified by spectrophotometric reading using a nanodrop at 260/280 nm wavelength, and DNA amplifiability was checked by qualitative PCR amplifying a conserved region in the bacteria (panB), as already described [34,39]. One µg of extracted DNA was then analyzed using the Microbial DNA qPCR Array for Antibiotic Resistance Genes (Qiagen, Hilden, Germany), allowing the simultaneous detection and quantification of 84 antibiotic resistance genes (ARGs), as previously described [34,35,36,37,39].

### 2.6. Statistical Analyses

Statistical analyses were performed using the GraphPad Prism 5.03 software (GraphPad Software, San Diego, CA, USA). The parametric Student’s *t*-test was used assuming as statistically significant a *p*-value of at least <0.05. To analyze the resistome data, the Bonferroni correction for multiple comparisons was applied to the value detected in the Student’s *t*-test, assuming a corrected pc value of 0.05 as statistically significant.

## 3. Results

### 3.1. School Environment Bioburden

To characterize the microbiological quality of the indoor school environment and evaluate the impact of green interventions aimed to improve it, a pre–post case–control study was performed in an Italian high school from March 2023 to May 2024, excluding the summer holiday period, for a 10-month total survey. Two classrooms with superimposable features were enrolled in the study, one receiving three experimental interventions (test room, TR) and the other not receiving any interventions and serving as a control (control room, CR). The study included four consecutive phases during which the interventions were implemented in the TR: (1) T0, providing basal values detected with usual conventional sanitation and ventilation via window opening; (2) T1, during which greenhouse plants were added to the TR; (3) T2, during which mechanical air ventilation was installed in the TR; and (4) T3, during which the TR received PCHS, probiotic-based sanitation, in substitution for the conventional chemical-based one. The classroom microbiome was monitored throughout the whole study via bimonthly sampling campaigns uniformly distributed during the study period. At each sampling campaign, five environmental samples were collected in duplicate from the air and surfaces (floor, student tables, and teacher’s desk), before and after the students’ classes. Surface points were simultaneously sampled by RODAC contact plates and swabs for microbiological culture-based analyses and PCR-based molecular analyses, respectively.

The results of the microbial monitoring were similar in the multiple sampling campaigns carried out within each period. Moreover, the environmental samples collected from the different surfaces (floor, student tables, desks) were considered together, since they expressed the overall contamination of the classroom environment. Based on this, the bioburden results were calculated and expressed as median values of those detected in the different sampling campaigns included in each period, showing the surface and air results separately.

**Figure 3:**
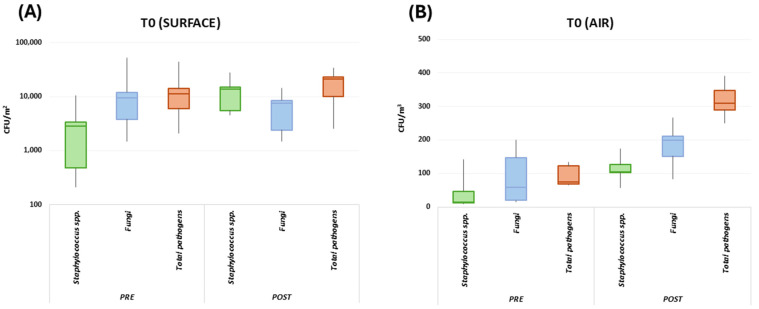
Basal level of pathogen bioburden (T0). Prevalent pathogens and the sum of all searched pathogens are shown. Contamination levels are illustrated as box plots with median, Q1, Q3, minimum, and maximum values of CFU/m^2^ or CFU/m^3^ detected in surface (**A**) and air (**B**) samples, before (pre) and after (post) lessons. Being superimposable, CR and TR values were considered together. *Staphylococcus* spp. are represented in green, fungi in blue and the total pathogens in red.

At the basal level (T0), the results collectively evidenced the presence of potential human pathogens in the classroom environment, as judged by RODAC CFU counts (Figure 3). The microbial bioburden, expressed as the sum of searched pathogens, included fungi (including *Aspergillus* spp.) and bacteria (mostly *Staphylococcus* spp., including *S. aureus*), and different levels of microbial contamination were detected on surfaces and in the air, and at pre- and post-lesson timepoints. No significant differences were observed between the TR and CR in any sample at T0 (*p* = n.s.); thus, a median value was collectively calculated for both enrolled classrooms.

In detail, before lessons, the T0 surface bioburden corresponded to 12,421 CFU/m^2^ (median value, range 3098–45,236 CFU/m^2^). After the lesson period (6 h), the surface contamination increased as expected, likely due to the presence of students, reaching 21,412 CFU/m^2^ (median value, range 2540–33,947 CFU/m^2^) (Figure 3). Besides the expected quantitative differences attributable to the presence of human occupants, qualitative differences were also observed in the bioburden composition before and after lessons. In detail, before lessons, the surface bioburden essentially consisted of fungi (76.7% of the total surface population, corresponding to a median value of 9526 CFU/m^2^), followed by *Staphylococcus* spp. (23.2% of the total surface bioburden, corresponding to a median value of 2881.7 CFU/m^2^). After lessons, fungi were not any more prevalent on surfaces, representing 35.6% of the total pathogens (7622.7 CFU/m^2^, median value). Instead, Staphylococci increased, accounting for 64.4% of the total bioburden (corresponding to a median value of 13,789.3 CFU/m^2^).

The contamination of the air was significantly lower since the total airborne bioburden corresponded to 75 CFU/m^3^ (median value, range 65–134 CFU/m^3^) before lessons and 310 CFU/m^3^ (median value, range 250–391 CFU/m^3^) after lessons (Figure 3). The airborne population was mainly attributable to fungi before and after lessons. Of note, *Aspergillus* spp. were detectable in air samples, though at a low level (0.01% of the total mycetes), highlighting the presence of this important human pathogen in this community environment. Overall, mycetes represented 76.5% of the total microbial population before lessons (corresponding to a median value of 57.4 CFU/m^3^) and represented 64% of the total airborne microbes after lessons (corresponding to a median value of 198.4 CFU/m^3^). *Staphylococcus* spp. were also well represented in the airborne microbial population, accounting for 19.8% of the total airborne pathogens before lessons (14.9 CFU/m^3^, median value) and 33.7% after lessons (104.5 CFU/m^3^, median value). Neither bacteria of the Enterobacteriaceae family nor mycetes of the *Candida* genus were detected at T0 in surface or air samples of the TR or CR (median value 0 CFU/m^2^, range 0–0 CFU/m^2^).

**Figure 4:**
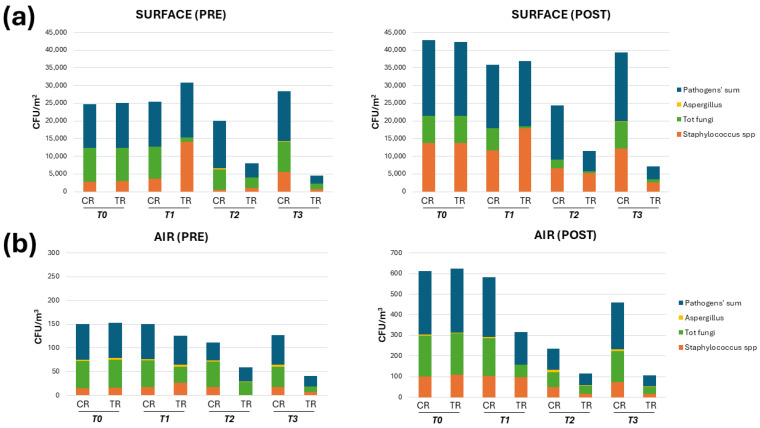
Pathogen bioburden in the CR and TR rooms. The presence and proportion of total and individual pathogens are shown during all the study periods (T0, T1, T2, and T3). The results are expressed as the sum of the median values obtained by RODAC sampling and CFU counts on surfaces (**a**) and in the air (**b**) before (pre) and after (post) lessons.

At T1 (Figure 4), no significant variations were observed in the control CR compared to the basal values detected at T0 (CR_T1_ vs. CR_T0_, *p* = n.s.). The total surface bioburden corresponded in fact to 12,689 CFU/m^2^ before lessons and 17,898 CFU/m^2^ after lessons. As observed at T0, before lessons, the surface microbial community was mainly represented by fungi (71.5%, 9072.6 CFU/m^2^), followed by Staphylococci (28.5%, 3616.4 CFU/m^2^), whereas after lessons, Staphylococci became prevalent (64.9%, 11,615.8 CFU/m^2^) and fungi decreased in relative abundance (35%, 6264.3 CFU/m^3^). Also, the total airborne bioburden levels were very similar to those observed at T0, corresponding to 74 CFU/m^3^ before lessons and 290 CFU/m^3^ after lessons. Fungi were prevalent in the air both before (75.1%, 55.6 CFU/m^3^) and after lessons (63.6%, 184.4 CFU/m^3^), as also detected at T0.

By contrast, some microbial alterations were observed in the TR, following the placement of green plants (T1). First, an overall increase in bioburden on surfaces was observed compared to T0 (15,473.7 vs. 12,421 CFU/m^2^), which was mainly attributable to a higher presence of Staphylococci, whose amount corresponded to a median value of 14,130.2 CFU/m^2^ (compared to 2881 CFU/m^2^; *p* < 0.001), thus representing the vast majority of surface bioburden even before lessons (91.3%). After lessons, the total surface bioburden further increased, with 18,549 CFU/m^2^ (median value), and *Staphylococcus* spp. represented 96.6% of the total microbial population (17,918.3 CFU/m^2^, median value). Fungi thus represented a very small fraction of the surface bioburden, both before (7.8%; 1206.9 CFU/m^2^) and after lessons (2.5%; 463.7 CFU/m^2^).

In contrast, the total airborne contamination appeared reduced in the TR at T1 compared to the control values both before lessons (61 CFU/m^3^ vs. 74 CFU/m^3^; *p* = n.s.) and after lessons (157.5 CFU/m^3^ vs. 290 CFU/m^3^; *p* < 0.05). Of note, the fungal component was particularly affected, representing only 56% of the total bioburden before lessons, corresponding to 34 CFU/m^3^ (instead of 71.5% and 55.6 CFU/m^3^ for the control). After lessons, the relative abundance of fungi was further decreased, as it represented 36.9% of the total bioburden, corresponding to 58.1 CFU/m^3^, compared to 184.4 CFU/m^3^ for the control. The introduction of green plants in the TR was thus associated with a whole reduction in fungal contamination corresponding to 38.8% (*p* < 0.05) before lessons and 68.4% (*p* < 0.01) after lessons, in comparison with the control CR values.

At T2, a mechanical ventilation system was installed in the TR, allowing a constant flow rate of 440 m^3^/h of fresh air (the same flow rate for the expelled air) during lesson time. The presence of mechanical ventilation kept temperature and humidity levels constant, at 21–23 °C and 40–45%, respectively, whereas in the CR, the temperature and humidity fluctuated continuously, increasing up to 25 °C and 65%, respectively. The microbial monitoring evidenced a clear reduction in pathogens associated with this intervention. Specifically, before lessons, the TR surface bioburden corresponded to 3998.9 CFU/m^2^ (median value; range 1052.63–4578.94 CFU/m^2^), compared with 13,368.4 CFU/m^2^ detected in the CR (median value; range 2549–25,621.5 CFU/m^2^), with a significant 70% reduction (*p* < 0.01). After lessons, the median value of surface contamination in the TR corresponded to 5789.5 CFU/m^2^ (median value, range 3578.9–6889.5 CFU/m^2^), compared with the 15,263.2 CFU/m^2^ measured in the CR (median value; range 6521.7–23,121 CFU/m^2^), confirming that the introduction of mechanical ventilation significantly reduced the surface contamination even in the presence of students (−62.1%; *p* < 0.05) (Figure 4).

The relative abundance of the main microbial components was similar to that observed at T0, despite the continuous presence of plants. Namely, in the TR, fungi were prevalent before lessons, representing 73% of total surface pathogens (2919.2 CFU/m^2^), whereas Staphylococci became prevalent after lessons (89%) (5152.7 CFU/m^2^). A clear decrease was also observed in the airborne bioburden in the TR compared to the control. In detail, the TR air bioburden corresponded to 28.5 CFU/m^3^ before lessons (median value, range 21–32 CFU/m^2^) and 58 CFU/m^3^ after lessons (median value, range 45–65 CFU/m^3^), whereas it corresponded to 38 CFU/m^3^ before lessons (range 29–61 CFU/m^3^) and 101 CFU/m^3^ after lessons (range 75–221 CFU/m^3^) in the CR. By comparing the TR and CR values, the airborne bioburden decreased by 26% at pre-lesson and by 42.63% at post-lesson timepoints (*p* < 0.05). In the air of the TR, fungi remained prevalent both before (93%) and after lessons (66.6%), as also observed in the CR environment, though the CFU number was diminished (26.5 vs. 52.2 CFU/m^3^ and 38.6 vs. 70.7 CFU/m^3^ in TR vs. CR values before and after lessons, respectively).

**Figure 5:**
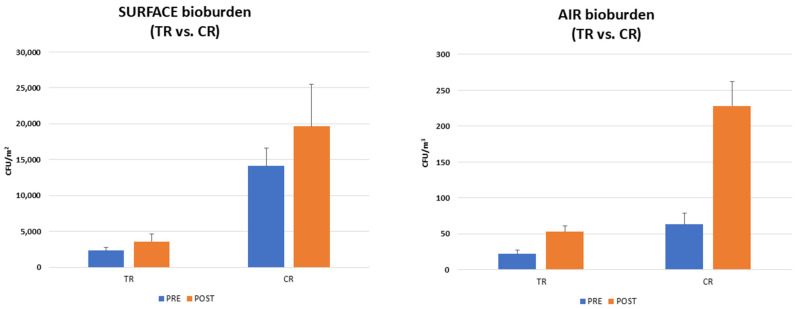
T3 surface and air bioburden in the TR and CR rooms. Results are expressed as median values of CFU/m^2^ ± SD (surface) and CFU/m^3^ ± SD (air) detected before (pre) and after (post) lessons.

At T3, probiotic-based sanitation (PCHS) was implemented in the TR in substitution for the conventional chemical one. As expected, based on previous results, this intervention was associated with significant changes in the TR environmental bioburden compared to the control (Figure 5). In the CR, surface contamination was in line with what was observed at the previous timepoints, corresponding to 14,105 CFU/m^2^ before lessons (median value; range 11,256–28.456.3 CFU/m^2^) and 19,631.8 CFU/m^2^ after lessons (median value; range 12,589.6–31,102 CFU/m^2^). Fungi were prevalent before the arrival of students (60% of total surface bioburden, corresponding to 8463 CFU/m^2^), whereas Staphylococci became prevalent afterward (62%; 12,173.6 CFU/m^2^). In contrast, the levels measured in the TR showed a significant decrease in all surface pathogens, which corresponded to 2315.8 CFU/m^2^ before lessons (median value, range 1473.7–4263.2 CFU/m^2^) and 3578.9 CFU/m^2^ (median value, range 1136.8–5105.3 CFU/m^2^) after lessons. The TR values were thus diminished compared to the CR ones, with 83.5% before lessons and 81.8% after lessons (*p* < 0.0001). On the other hand, the proportions of different microbial components were maintained, with fungi representing 68% of the total surface bioburden before lessons (1574 CFU/m^2^, median value) and Staphylococci becoming prevalent after lessons (75%; 2684 CFU/m^2^, median value). The decrease in pathogenic bioburden was paralleled by a gradual increase in PCHS-derived *Bacillus* spp., which reached 7578.9 CFU/m^2^ at the end of the T3 period, thus representing 76.6% and 67% of the total microorganisms detected in the classroom before and after lessons, respectively.

Also, air contamination levels remarkably decreased in comparison with the CR, since the TR air pathogens corresponded to 22 CFU/m^3^ before lessons (median value, range 9–69 CFU/m^3^) and 53 CFU/m^3^ after lessons (median value, range 15–81 CFU/m^3^). In contrast, in line with previous periods, the CR exhibited 63 CFU/m^3^ of airborne pathogens before lessons (median value; range 18–101 CFU/m^3^) and 228 CFU/m^3^ after lessons (median value; range 141.2–352 CFU/m^3^). Fungi were prevalent both before and after lessons in the CR (68% and 66.8%, respectively) and in the TR, where they represented 51% and 64% of total airborne pathogens before and after lessons, respectively. Of note, while *Aspergillus* was detected in the CR, with a median value of 3 CFU/m^3^ (range 0–5 CFU/m^3^), no *Aspergillus* was found in the TR (median value 0 CFU/m^3^, range 0–0 CFU/m^3^). Based on the measured CFU numbers, the decrease in air contamination was 65.1% before lessons and 76.8% after lessons (*p* < 0.001). Figure 5 specifically evidences the differences between TR and CR bioburden levels at the final T3 study period.

### 3.2. Resistome Characterization of the Classroom Bioburden

Floor samples, being the most abundant in terms of microbial load, were also analyzed by qPCR microarray to profile the antibiotic resistance genes (ARGs) harbored by the microbial population persistently colonizing the classroom environment. The results evidenced a low but detectable presence of different ARGs in the classroom microbes, conferring resistance against antibiotics belonging to different classes, including aminoglycosides, tetracycline, beta-lactams (including methicillin), and macrolides. Most detected ARGs and their functions are summarized in Table 2.

**Figure 6:**
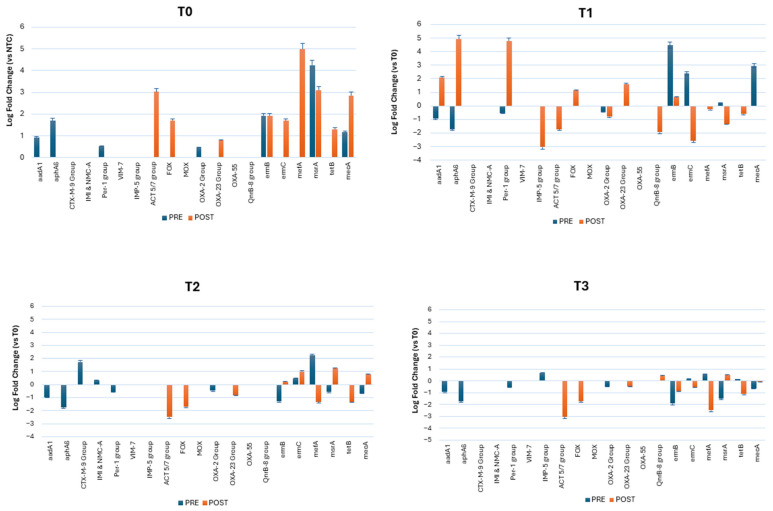
Resistome characterization in the TR classroom. ARGs were evidenced by qPCR microarray, performed on floor samples at the indicated times (T0, T1, T2, and T3) before (pre) and after (post) lessons. Results are expressed as mean values ± SD of the Log_10_ fold change (FC) for every ARG. T0 values were obtained for comparison with the negative control (NTC) values, whereas T1, T2, and T3 values were obtained for comparison with the T0 values, as indicated on the *y*-axis.

In the CR, the abundance of ARGs detected at T0 remained nearly unchanged throughout the whole study, whereas some noteworthy variations were observed in the TR following the introduction of the planned interventions (Figure 6). At T0, both the TR and CR showed the same ARGs, without any relevant differences. The prevalent ARGs before lessons included, in order of abundance (expressed as Log_10_ fold change, FC, with respect to the negative control, NTC), msrA (4.2 Log_10_ FC), ermB (1.9 log_10_ FC), aphA6 (1.7 Log_10_ FC), and mecA (1.16 Log_10_ FC), followed by aadA1 (0.9 Log_10_ FC), Per-1 group (0.5 Log_10_ FC), and OXA-2 group (0.45 Log_10_ FC). *S. aureus* and its virulence genes spa and luk (included in the microarray) were also detected at high frequency (4.47 Log_10_ FC). Notably, mecA was found in the classroom environment, evidencing the likely presence of MRSA (methicillin-resistant *S. aureus*) in the school environment. After the attendance of students, further ARGs appeared, likely as a consequence of the spread of students’ bacteria. The most prevalent ARGs after lessons included mefA (5 Log_10_ FC), msrA (3.1 Log_10_ FC), ACT 5/7 group (3.03 Log_10_ FC), ermB (1.9 Log_10_ FC), FOX (1.7 Log_10_ FC), ermC (1.7 Log_10_ FC), and tetB (1.3 Log_10_ FC).

At T1, following plant introduction, a decrease in some ARGs was observed in the TR, whereas ermB (4.5 Log_10_ FC) and mecA (2.9 Log_10_ FC) appeared slightly increased, probably as a consequence of the increased number of Staphylococci observed in the TR during the T1 phase of the study. No statistical significance was detected in any of the observed differences. After classes, compared to what was observed at T0, some ARGs were decreased up to 2 Logs (ACT 5/7 group, FOX, ermB, mefA), but new ARGs appeared, including aphA6 (2.08 Log_10_ FC), CTX-M-9 group (4.9 Log_10_ FC), and VIM-7 (4.7 Log_10_ FC).

At T2, upon the installation of a mechanical ventilation system, the number of ARGs appeared to decrease compared to T0, both before and after lessons, likely in association with the decreases observed in the whole bioburden by CFU counts. However, while aadA1, aphA6, and ermB were decreased by up to −1.5 Log_10_ FC before lessons, CTX-M-9 group and mefA were increased by around 2 Log FC compared to T0. After lessons, a remarkable drop in almost all ARGs detected at T0 was recorded, except for ermC, msrA, and mecA, which were increased by about 1 Log_10_ each.

In contrast, at T3, the introduction of PCHS sanitation was associated with an overall decrease in all the ARGs detected at T0, T1, and T2 before lessons (up to −99.9%, *p_c_* < 0.001). The decrease was also evident after lessons, when all the ARGs identified in previous periods appeared to be significantly diminished (up to −3 Log_10_), thus confirming the significant impact of probiotic-based sanitation in preventing the diffusion of drug-resistant microbes in both sanitary and non-sanitary environments.

**Figure 7:**
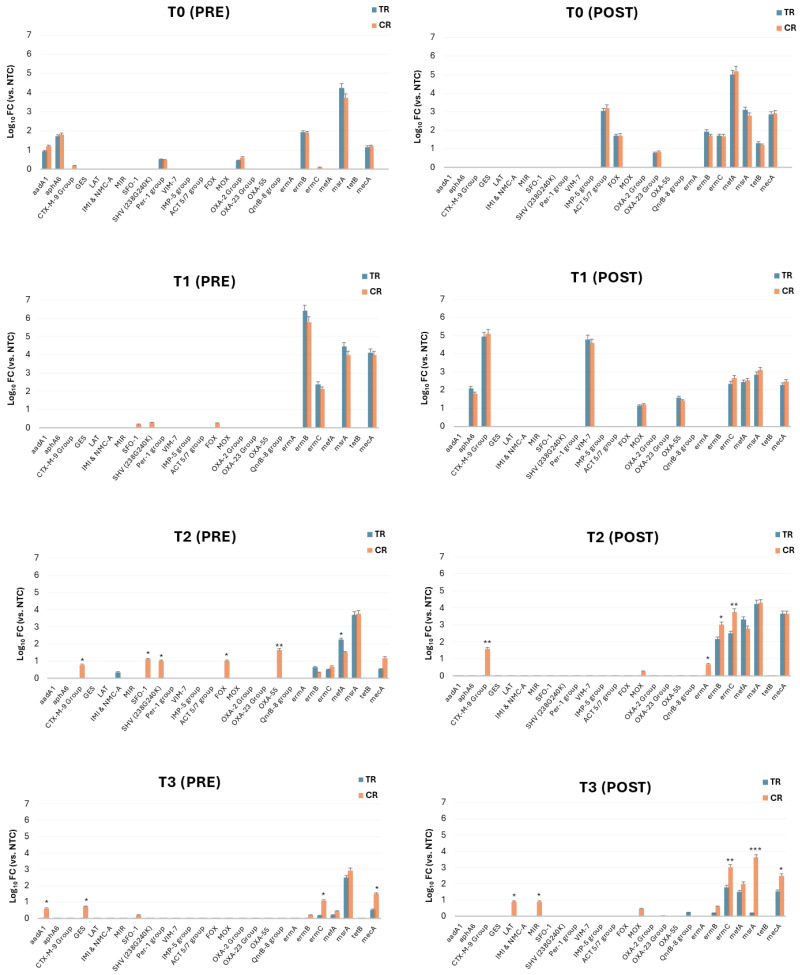
Resistome characterization in the TR and CR rooms. ARGs were quantified by qPCR microarray performed on floor samples at the indicated times (T0, T1, T2, and T3) before (pre) and after (post) lessons. Results are expressed as mean values ± SD of the Log_10_ fold change (FC) vs. the NTC for every indicated ARG. Asterisks indicate statistically significant differences between TR and CR values (*, *p_c_* < 0.05; **, *p_c_* < 0.01; ***, *p_c_* < 0.001).

Of note, while the differences observed between the TR and CR were not significant at T0 and T1, they became statistically significant at both T2 and T3 (0.05 < *p_c_* < 0.001) (Figure 7). In particular, at T3, the differences were significant for all the ARGs detected at both pre- and post-lesson timepoints. Similarly, the presence of virulent *S. aureus* (associated with the virulence genes spa and luk F) appeared to be significantly diminished only at T3, in the TR vs. the CR.

## 4. Discussion

Among high-traffic BEs, schools represent the one where humans spend most of their time during the educational phase of their lives. Of note, microbes have been persistently detected on surfaces and in the air of the school environment, where they have been associated with the onset of various diseases in this age group [1,11,12,18]. Consequently, monitoring the school microbiome may be important to maintaining a healthy learning environment and preventing the onset of diseases in students and teaching staff. It is recognized that indoor microbiomes are affected by ventilation type, building features, and cleaning modalities. Inadequate airflow is in fact associated with health complications [14,69], and plant introduction is considered a possible tool for improving air quality and cognitive performance in students [70,71]. Moreover, bioburden control has so far been addressed by conventional chemical disinfection, which may increase chemical pollutants and induce the potential selection of drug-resistant microbes [72,73,74]. In contrast, sustainable cleaning systems based on probiotic use (PCHS) have been reported as a promising alternative, having been shown to stably control bioburden without selecting AMR [34,35,36,37,39,40,42,43,75].

In this study, we thus investigated the effects of different interventions (plant introduction, mechanical ventilation systems, and PCHS sanitation) on the school microbiome by performing a pre–post case–control study in an Italian high school.

The results collected at the basal level in both enrolled classrooms (TR and CR) showed the presence of several potential pathogens both on surfaces and in the air. The whole surface bioburden appeared consistent with that measured in non-sanitary environments [76,77], with >12,000 CFU/m^2^ before the entrance of students and around 20,000 CFU/m^2^ after six hours of the continuous presence of occupants (students and teachers). Fungi and Staphylococci were the prevalent microbes, in line with what was previously observed. Fungi accounted for over 75% of the surface bioburden before lessons and *Staphylococcus* spp. represented >60% of the total bioburden measured after lessons. Of note, *Aspergillus* species were almost always detected as part of the resident fungal population, raising potential risks for the health of human occupants. In fact, some *Aspergillus* species can be harmful to humans, as they behave as significantly opportunistic pathogens, potentially causing multiple diseases, including invasive pulmonary infections and allergic diseases, especially in fragile subjects [78]. These results are in line with those obtained in other studies, particularly in school areas with inadequate ventilation and high humidity [79,80,81], and further support the fact that human-derived bacteria are common in spaces where people (such as students) congregate [82,83].

Of note, several genes conferring antibiotic resistance (ARGs) were identified in the microbial population persisting in the classroom environment, confirming that AMR is no longer confined to hospitals but is spreading significantly outside of sanitary settings. Prevalent ARGs included genes conferring resistance against different classes of antibiotics, including beta-lactams, aminoglycosides, and macrolides. Methicillin resistance, likely ascribable to the presence of methicillin-resistant *S. aureus* (MRSA) [84], was also detected. This finding further highlights the spread of MRSA in the general population in non-sanitary environments, and is in line with previous studies performed on antibiotic-resistant microbes in schools [85,86,87] and in high-traffic community environments [35].

The introduction of *Golden pothos* and *Tillandsia kammii Rauh* plants, chosen essentially for their resilience and suitability for greening walls, caused an unexpected shift in the TR microbial population, with a significant increase in the staphylococcal component and a concomitant reduction in the fungal one. This may be attributed to the potential impact of plants on the classroom’s microbial dynamics via changes in environmental factors able to slow fungal proliferation or through the release of bacterial species from the plant microbiome, in line with findings obtained in a previous study [20]. More specifically, changes in physical parameters (such as temperature and relative humidity), as well as the release of volatile organic compounds (VOCs) and other plant-derived factors, may have created an environment favoring the growth of Staphylococci but not that of fungi, which typically thrive in more humid conditions [88]. Also, specific plant-associated bacteria may have outcompeted fungi and contributed to the observed increase in *Staphylococcus* spp. This hypothesis appears consistent with previous studies showing that plant-associated bacteria could effectively colonize indoor environments [20,89] and compete with fungi for nutrients and space. However, since comprehensive species identification of all the bacterial species was not performed, it is not currently possible to determine whether the increased bacteria were environmental Staphylococci or cocci of human origin, and more detailed analyses should be performed to further explore this aspect (including whole-genome sequencing, WGS) and clarify the sources of dynamics of these microbial populations.

The installation of mechanical ventilation systems is instead recognized as a potential factor able to significantly impact the indoor microbiome, and consistent with this at T2, a significant reduction in contamination was observed in the TR compared to the CR. More specifically, a drop of 70% and 62% was observed in the total microbial population measured before and after lessons, confirming that mechanical ventilation can efficiently prevent over-contamination, likely by controlling the environmental factors that could favor the growth of pathogens, such as temperature and humidity [90]. All the details of the ventilation systems adopted during the study are described in a companion article [91]. Noticeably, during the first phase, room ventilation was obtained by opening windows and doors, resulting in a highly variable rate over time, also depending on external climatic conditions and the amount of time the windows were open. With the introduction of mechanical ventilation, the airflow rate was set at 440 m^3^/h, equivalent to 24 m^3^/h per person (17 students and 1 teacher—6.7 L/s per person), in accordance with the European national standards (UNI EN 16798-1) [92], which require a minimum air exchange of 4 L/s (14.4 m^3^/h) per person in schools. The records of CO_2_, temperature, and relative humidity showed that relative humidity was significantly lower in the TR, where it did not exceed 45%, compared to the CR, where it constantly reached 65%. This parameter, together with the temperature (maintained at 21–23 °C), may have significantly impacted the growth of microorganisms inside the TR. Moreover, the accurate monitoring of CO_2_ concentration [91] showed that CO_2_ concentrations dropped significantly in the TR with mechanical ventilation (<1300 ppm-v) compared to natural ventilation (4500 ppm-v), with an average decrease in concentration of 62%, with potential improvement in psychological well-being and learning outcomes.

At T3, further significant changes were observed, associated with the introduction of PCHS in substitution for conventional chemical cleaning, based on the use of denatured alcohol on furniture surfaces and chlorine on the floors. The results, in fact, evidenced a significant drop in surface pathogens of 83% before lessons and of 81% after lessons, compared with what was detected in the CR (*p* < 0.001). PCHS, by introducing beneficial *Bacillus* probiotics, has previously been recognized as an effective tool to stably control the colonization by pathogens in treated environments [27,34,36,37,39,42,43,93,94] via active competition with the surrounding pathogens. Our data confirm the results previously obtained in other community spaces [35], showing a significant stabilizing effect of PCHS both on the surface and in the air both before and after lessons, associated with the increase in *Bacillus* in the treated environment. In particular, the low number of pathogens detected after lessons highlights PCHS’ ability to prevent recontamination of the treated environment, despite the continuous microbial spread associated with student occupancy. These data thus support the usefulness of PCHS in high-traffic non-sanitary settings to protect the environment long term from potentially harmful contamination [34,35,36,37,39,42,43,75]. Moreover, considering that the abundance of indoor Bacilli has been significantly associated with gut microbial diversity and the Gut Microbiome Health Index [95], these data may also be of importance toward improving the general health of attending students. In addition, the resistome analysis showed that the application of PCHS was associated with an overall decrease in all the ARGs detected in the classroom environment of up to 3 Log_10_ in the previous study periods, confirming that it may be an important tool for tackling the spread of AMR in the community. Thus, probiotic cleaning was compatible with the presence of a mechanical ventilation system (which did not disturb the PCHS–*Bacillus* colonization) and could further contribute to the decrease in pathogens and AMR in a summation mode. Our school microbiome data are in line with those reported in previous case studies, although a direct comparison is difficult because most published data derive from NGS analyses, whereas we quantified the microbial contaminants by CFU count. Despite these methodological differences, Staphylococci and other human-derived bacteria were reported as the main source of bacteria in classrooms, together with fungi mostly attributed to the outdoor environment [96]. Of note, the bacterial/fungal fraction of the school indoor microbiome was associated with respiratory diseases [97,98], whereas increased biodiversity in the school microbiome was instead suggested to protect students and school staff against these infections [97]. Various studies have shown that the presence of green plants is associated with an increased number of bacterial taxa [99], and further research would be needed to analyze in detail the plant-related taxa composition of the indoor microbiome and to explore its contribution to health effects. Other studies have confirmed that indoor plants can enrich indoor microbial diversity [100] and support the enhancement in commensal skin microbes and immune regulation [101]. Last, green plants were also associated with 10% lower CO_2_ and more stable temperatures [102].

Ventilation was also reported to affect surface and airborne bacteria and fungi possibly associated with respiratory symptoms in schoolrooms [103], and to impact CO_2_, temperature, and humidity parameters of the indoor air [104], thus strengthening what we observed. No studies on PBS usage in the indoor school environment are available in the literature, but several studies were conducted in sanitary and non-sanitary areas, showing a significant decrease in potential surface and airborne pathogens [27,34,35,36,37,39,42,43,93]. Major published school case studies are summarized in Table 3 and emphasize the overall importance of understanding the interaction between the indoor microbiome and the environmental characteristics (plants, relative humidity, building confinement, and CO_2_ concentration) in designing disease prevention strategies [3].

Our data show that different types of sustainable intervention can be compatible with each other and concur to obtain additive results, exceeding those achievable by applying separate interventions. A combined intervention including plants, mechanical ventilation, and probiotic cleaning could thus be highly effective at reducing the indoor bioburden and AMR, suggesting that an integrated approach may not only help to reduce all persistent pathogens but also have a direct impact on limiting the presence and spread of antibiotic-resistant microorganisms in these environments, which is particularly important in public spaces, where exposure to resistant strains can pose a serious health risk to vulnerable populations such as students.

This study was originally designed as a proof of concept and suffers from some limitations that cannot currently support high generalizability of the collected results, including (1) the sample size: this interventional study was conducted in a single school, and the results should be confirmed in other schools with diverse geographical locations, building types, ventilation systems, seasonal variations, and student populations; (2) the short study periods, particularly periods T1 (only plants) and T2 (mechanical ventilation), since this limited timeframe may not have been sufficient to fully observe the long-term effects and stabilization of the microbial community following these specific interventions, with the short duration of phases T1 and T2 being primarily due to logistical needs linked to the development of the PTSO project within the school timing constraints, and with the understanding that extending the intervention periods could provide more robust data; and (3) the type of analysis: CFU count and qPCR microarray were used with the aim of detecting any changes in pathogen and ARG amounts, but a comprehensive analysis of the whole microbiome performed by NGS or WGS would be important to profile the whole microbial population and assess its features, such as biodiversity. High-throughput sequencing techniques would in fact overcome the limitations linked to the usage of culture-based techniques, providing a comprehensive picture of the indoor school microbiome, which may reinforce and confirm our data, be useful in comparison with reported case studies to assess the generalizability of the observed effects, and allow for less speculative conclusions. Indoor microbiome studies may in fact provide precise identification of health-related targets and contribute to optimizing standard practices.

## 5. Conclusions

The present study represents a part of a Path for Transversal Skills and Orientation (PTSO) project about sustainability, performed with students at an Italian high school. Despite the study limitations (monocentric, limited time period, lack of NGS analysis), the collected results show that a combined strategy aimed at improving the indoor microbiome could be effective in controlling pathogen contamination, AMR diffusion, and environmental parameters (CO_2_, temperature, humidity) in the school areas. The sustainable interventions adopted in the test classroom included the addition of plants, mechanical ventilation, and probiotic cleaning, and overall evidenced a significant modification in the indoor surface and air microbiome, which may provide a healthier environment for students and staff. Overall, the data suggest that the use of a holistic approach taking into consideration all the parameters affecting the quality of indoor spaces could sustainably and effectively reduce the infectious risk for human occupants and the spread of AMR, meanwhile preserving the health of the outdoor environment, in line with the One Health principles. Further research based on larger samples, including diverse school building types and geographical locations, as well as the application of different methodologies (including deep sequencing molecular methods), could help to achieve a better understanding of the microbial dynamics within the school environment.

## Figures and Tables

**Table 1:** Main features of the enrolled classrooms.

Features	Measures
Length	8.47 m
Width	5.75 m
Ceiling height	3.23 m
Maximum height	5.15 m
Floor area	48.7 m^2^
Volume	170 m^3^

**Table 2:** Most detected ARGs with encoded functions.

ARGs	Antibiotic/Gene Function	Reference
*aadA1*	Aminoglycoside	Hollingshead et al., 1985 [44]
*aphA6*	Aminoglycoside	Aris et al., 2019 [45]
*CTX-M-9 Group*	Class A beta-lactamase	Sun et al., 2010 [46]
*GES*	Class A beta-lactamase	Lee et al., 2005 [47]
*IMI & NMC-A*	Class A beta-lactamase	Walther-Rasmussen et al., 2007 [48]
*SFO-1*	Class A beta-lactamase	Matsumoto et al., 1999 [49]
*SHV (238G240K)*	Class A beta-lactamase	Caselli et al., 2016 [39]
*Per-1 group*	Class A beta-lactamase	Aly et al., 2016 [50]
*VIM-7*	Class B beta-lactamase	Toleman et al., 2004 [51]
*IMP-5 group*	Class B beta-lactamase	Brízio et al., 2006 [52]
*ACT 5/7 group*	Class C beta-lactamase	Guan et al., 2024 [53]
*FOX*	Class C beta-lactamase	Gonzalez Leiza et al., 1994 [54]
*LAT*	Class C beta-lactamase	Tzouvelekis et al., 1994 [55]
*MIR*	Class C beta-lactamase	Papanicolaou et al., 1990 [56]
*MOX*	Class C beta-lactamase	Oguri et al., 2014 [57]
*OXA-2 Group*	Class D beta-lactamase	Bhattacharjee et al., 2015 [58]
*OXA-23 Group*	Class D beta-lactamase	Smith et al., 2013 [59]
*OXA-55*	Class D beta-lactamase	Héritier et al., 2004 [60]
*QnrB-8 group*	Fluoroquinolone	Rezazadeh et al., 2016 [61]
*ermA*	Macrolide lincosamide streptogramin_b	Malhotra-Kumar et al., 2009 [62]
*ermB*	Macrolide lincosamide streptogramin_b	Min et al., 2008 [63]
*ermC*	Macrolide lincosamide streptogramin_b	Shivakumar et al., 1981 [64]
*mefA*	Macrolide lincosamide streptogramin_b	Daly et al., 2004 [65]
*msrA*	Macrolide lincosamide streptogramin_b	Poole et al., 2005 [66]
*tetB*	Tetracycline efflux pump	Warburton et al., 2013 [67]
*mecA*	Methicillin	Utsui et al., 1985 [68]

**Table 3:** Major published studies on the indoor microbiome in community environments.

Setting	Study Aim	Analysis Methods	Primary Outcomes	References
Elementary schools (Korea)	Microbial monitoring	NGS (16S rRNA/ITS)	Bacteria derived from humans Fungi mostly attributed to the outdoor environment	Lee et al., 2021 [96]
Elementary schools (USA)	Impact of indoor microbiome on resp. infections	NGS (16S rRNA/ITS)	Indoor microbiome with high biodiversity may provide better protection against respiratory infections	Park et al., 2025 [97]
Dormitory rooms (China)	Impact of indoor microbiome on rhinitis	NGS (16S rRNA)	Indoor microbiome associated with rhinitis (specific taxa)	Fu et al., 2024 [98]
155 Households (Belgium)	Impact of plants on indoor microbiome	NGS (16S rRNA/ITS); qPCR	Indoor plants associated with increased microbial biodiversity. Bacterial/fungal richness increased with >3 plants	Dockx et al., 2022 [99]
176 Living rooms (Belgium)	Impact of outdoor green space on indoor microbiome	NGS (16S rRNA/ITS); qPCR	Residential green associated with increased microbial biodiversity	Dockx et al., 2021 [100]
Offices (Finland)	Impact of green walls on skin microbiota and immunity	NGS (16S rRNA)	Air-circulating green walls may enhance skin health and immune response.	Soininen et al., 2022 [101]
Secondary school (Sweden)	Impact of indoor plants on environment	Measurement of indoor physical parameters (sensors)	Indoor plants associated with decreased CO_2_ (−10%) and more stable temperature	Danielski et al., 2022 [102]
Several schools	Review of indoor air quality and health	Correlation of ventilation, CO_2_, and CFU counts	Bacterial/fungal pathogens in classroom air Inadequate ventilation decreases air quality (CO_2_ and bioburden).	Daisey et al., 2003 [103]
High school (Italy)	Environmental and microbial monitoring	Measurement of CO_2_ and CFU counts in indoor air	*Staphylococcus* spp. and other potential pathogens found in indoor air Inadequate ventilation affects air quality (CO_2_).	Langiano et al., 2024 [104]

## Data Availability

The original contributions presented in this study are included in the article. Further inquiries can be directed to the corresponding authors.
